# Implementing an intensified mentorship approach towards accelerated medical laboratory accreditation in 10 counties in Kenya

**DOI:** 10.4102/ajlm.v11i1.1814

**Published:** 2022-07-01

**Authors:** Susan K. Musau, Christina Mwachari, Elvis Kirui, Junghae Muthoni, Taylor Lascko, Natalia Blanco, Alash’le Abimiku, Emily Koech

**Affiliations:** 1 Department of Laboratory, Maryland Global Initiatives Corporation (MGIC), Nairobi, Kenya; 2 Department of Strategic Information, Maryland Global Initiatives Corporation (MGIC), Nairobi, Kenya; 3 Laboratory Department, Centers for Disease Control, Nairobi, Kenya; 4 Center for International Health, Education, and Biosecurity, University of Maryland, Baltimore, Maryland, United States; 5 School of Medicine, University of Maryland, Baltimore, Maryland, United States; 6 Center for International Health, Education, and Biosecurity (CIHEB), Nairobi, Kenya

**Keywords:** accreditation, SLMTA, SLIPTA, QMS, intensified mentorship, Kenya

## Abstract

**Background:**

Despite Kenya’s roll-out of the Strengthening Laboratory Management Towards Accreditation programme in 2010, most laboratories had not made significant or tangible improvements towards accreditation by 2016. In April 2016, the University of Maryland, Baltimore enrolled 27 facilities in the standard Strengthening Laboratory Management Towards Accreditation programme.

**Objective:**

This study aimed to describe and evaluate the implementation of an intensified mentorship strategy on laboratory accreditation.

**Methods:**

In October 2017, the University of Maryland, Baltimore implemented intensive mentorship in 27 hospital laboratories in Nairobi, Kiambu, Meru, Embu, Muranga, Nyeri, Laikipia, Nyandarua, Tharaka-Nithi, and Kirinyaga counties in Kenya. Laboratories were paired with competent mentors whose skills were matched to facility gaps. Baseline and follow-up assessments were done between April 2016 and March 2019 using the World Health Organization’s Stepwise Laboratory Quality Improvement Process Towards Accreditation Checklist and overall scores of the 12 Quality System Essentials and star ratings (from zero to five, based on scores) used to evaluate the effectiveness of the intensified mentorship.

**Results:**

In September 2017, 14 laboratories scored zero stars, three scored one star, eight scored two stars, one scored three stars, and one laboratory was accredited. By March 2019, eight laboratories were accredited, five scored four stars, 10 scored three stars, three scored two stars, and only one scored one star. The average score change with the intensified approach was 81.5 versus 53.9 for the standard approach.

**Conclusion:**

The intensified mentorship strategy resulted in fast-tracked progress towards laboratory accreditation and can be adopted in similar resource-limited settings.

## Introduction

Implementing a laboratory quality management system (QMS) is critical to providing reliable results for clinical decision-making.^1^ Inaccurate laboratory results can lead to inappropriate interventions, adversely affecting the credibility of the laboratory, and may invite legal action.^2^ Laboratory accreditation formally recognises both the QMS and the technical competence of a laboratory to perform specific tests.^1,3^

Laboratory accreditation is a system of standard procedures that aims to improve laboratory services’ quality, results’ accuracy, and patients’ safety.^4,5^ Most laboratories rely on international quality standards for medical laboratories (International Organization for Standardization 15189), which specify the requirements of quality and competence to medical laboratories. This set of standards is comprehensive but also resource-intensive.^5^ Resource-limited settings face several challenges when implementing quality standards and practices in laboratories, including financial constraints, human resource capacity, limited physical infrastructure, lack of equipment and equipment maintenance, failure to create and/or implement national laboratory policies, and substandard performance in laboratory quality indicators due to a lack of quality standards.^6,7^

The Strengthening Laboratory Management Towards Accreditation (SLMTA) programme was launched in 2009,^8^ and adopted in Kenya in 2010. Strengthening Laboratory Management Toward Accreditation is a competency-based programme that uses a series of short courses and work-based learning projects to effect immediate and measurable laboratory improvements while empowering laboratory managers to implement a practical QMS to ensure better patient care. A standard SLMTA training programme spans from 12 to 18 months. It includes three workshops spaced between QMS’ application and improvement projects’ implementation. The process is supported by regular supervisory visits or on-site mentoring. Audits are conducted at the beginning, midterm, and end of the training using the World Health Organization’s Stepwise Laboratory Quality Improvement Process Towards Accreditation (SLIPTA) checklist to assess strengths, weaknesses, and progress made.^9^ Laboratories scoring three stars and above are eligible to apply to the Kenya Accreditation Service, the sole national accreditation body mandated to offer accreditation services for laboratories in Kenya.

Kenya has progressed tremendously in using the SLMTA programme to accredit medical laboratories, however, only 13 laboratories had been accredited by 2016 through the SLMTA programme.^10,11^ With more than 3000 medical laboratories in Kenya offering basic diagnostic services, a considerable gap remains in implementing QMS.

The University of Maryland, Baltimore (UMB), with funding from the United States President’s Emergency Plan for AIDS Relief through the United States Centers for Disease Control and Prevention (CDC), has worked with Kenya’s national and county governments to enhance laboratory systems to support HIV/tuberculosis services. As part of this scope, UMB, through the Boresha Maabara programme, supported and mentored 27 hospital laboratories from 10 selected counties (Nairobi, Kiambu, Meru, Embu, Muranga, Nyeri, Laikipia, Nyandarua, Tharaka and Kirinyanga) in implementing continuous quality improvement initiatives, and laboratory QMS mentorship towards accreditation. The hospitals laboratories included all cadres, such as county, districts and low-level health centres. The objective of this paper is to describe and evaluate an intensified laboratory QMS mentorship strategy, which was introduced by UMB to move public health laboratories in Kenya to accreditation.

## Methods

### Ethical considerations

The implementation protocol was approved by the University of Maryland, Baltimore Institutional Review Board (HP-00094192) and Amref Ethics and Scientific Review Committee (P 815/2020). This project was also reviewed in accordance with the CDC human research protection procedures. The Institutional Review Boards gave this protocol a “not human research” determination. Only aggregated non-human and non-identifiable data were used for this analysis.

### Progression of mentorship strategy

**FIGURE 1:**
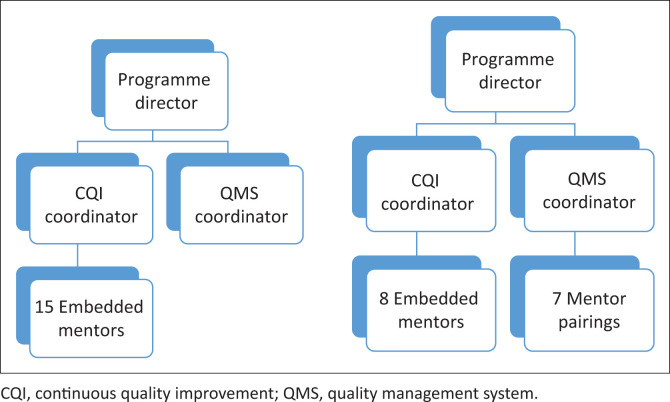
Comparison between the standard and intensified mentorship structures in University of Maryland, Baltimore from April 2016 to March 2019, Kenya.

At the Boresha Maabara programme’s inception in April 2016, 27 facilities were enrolled in the SLMTA programme. Baseline audits using the SLIPTA checklists were conducted in April 2016. Key healthcare workers, including laboratory managers and quality assurance officers, were enrolled in three consecutive QMS training workshops. Identified gaps were addressed through improvement projects, and their progress was evaluated during midterm audits. The standard SLMTA mentorship approach, as described by Makokha et al.,^12^ was used to support the facilities towards achieving accreditation, including embedding QMS mentors and supervisory mentorships in the laboratories for 360 days. Using this approach, 15 mentors were assigned to at least two SLMTA facilities. In addition to laboratory QMS mentorship, mentors were involved in supporting continuous quality improvement initiatives for HIV and tuberculosis testing-related services in other facilities. This broad scope of work stretched their workload and time (Figure 1).

The Boresha Maabara programme implemented an intensified mentorship approach, starting October 2017, to accelerate the accreditation process. In this approach, seven mentors were identified and dedicated purely to mentoring the 27 laboratories towards accreditation (Figure 1). The approach was overseen by a intensively trained QMS coordinator who had technical expertise and laboratory accreditation experience. The QMS coordinator led planning, supervising, and training facility teams on QMS initiatives. Mentorship involved conducting initial audits, reviewing non-conformities for each laboratory, and planning meetings for target setting and mentor pairing to support facilities based on non-conformities. Mentor competencies and strengths were matched with the laboratory needs, based on the 12 Quality System Essentials, and mentorship addressed the 12 Quality System Essentials for each laboratory to resolve all non-conformities.

Mentorship was complemented with International Organization for Standardization 15189:2012, method verification, root cause analysis/Corrective Action and Preventive Action, and safety trainings based on the laboratory’s needs. Monthly virtual meetings were held to check on the progress of all 27 laboratories, which involved re-strategising and re-identifying the skillsets required in different facilities, including re-assigning mentors to different laboratories, if needed. Internal audits were conducted by UMB mentors, and external audits by the African Society for Laboratory Medicine and Kenya Accreditation Service. The CDC engaged African Society for Laboratory Medicine to conduct external audits to map SLMTA laboratories in Africa, which allowed additional audits to be conducted before Kenya Accreditation Service assessments for accreditation.

### Data collection and analysis

**FIGURE 2:**
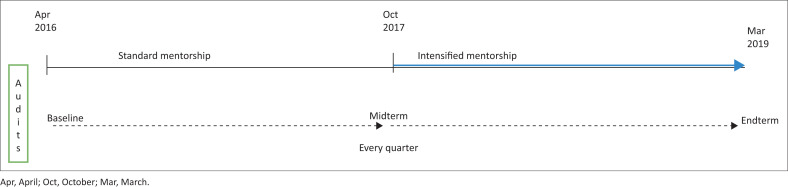
Timeline of the evaluation of 27 laboratories starting April 2016 to March 2019, Kenya.

Baseline and quarterly audits using the SLIPTA checklist (Figure 2) were conducted across the 27 laboratories, starting from April 2016 to March 2019, and scores were assigned based on standard scores allocated to the 12 SLIPTA sections. Data were analysed using descriptive statistics and graphical representations of the star ratings. Accredited laboratories were allocated the highest SLIPTA score (275) of a five-star rating during the computation. We calculated means, standard deviations, medians, and interquartile ranges for scores. The statistical analysis was performed using Stata Statistical Software Release 17 (College Station, Texas, United States: StataCorp LLC).

## Results

**FIGURE 3:**
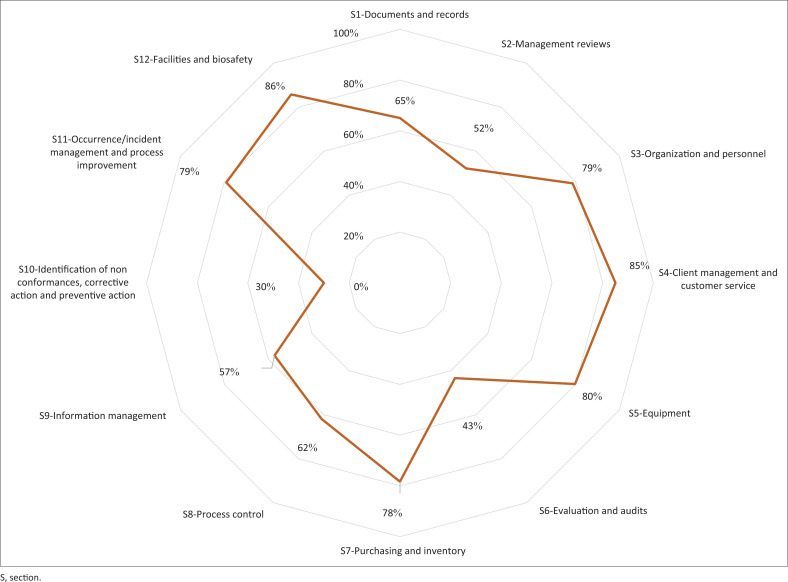
Spider chart showing mean scores for 10 University of Maryland, Baltimore-supported laboratories for Stepwise Laboratory Quality Improvement Process Towards Accreditation sections audited in September 2017, Kenya.

At baseline in April 2016, 23 laboratories scored zero stars, three scored one star, and one was already accredited. After 18 months of implementation of the standard mentorship strategy (April 2016 – September 2017), 14 laboratories scored zero stars, three scored one star, eight scored two stars, one scored three stars and one laboratory was accredited, showing slow improvement. In September 2017, the midterm audit demonstrated the common non-conformities emanated from three SLIPTA sections: Management Review Meetings (Section 2), evaluation and audits (section 6), and identification and control of non-conformities (section 10) (Figure 3). These non-conformities were addressed throughout the intensified mentorship through targeted trainings on how to convene and conduct Management Review Meetings, root cause analysis, corrective actions, and internal audits.

**FIGURE 4:**
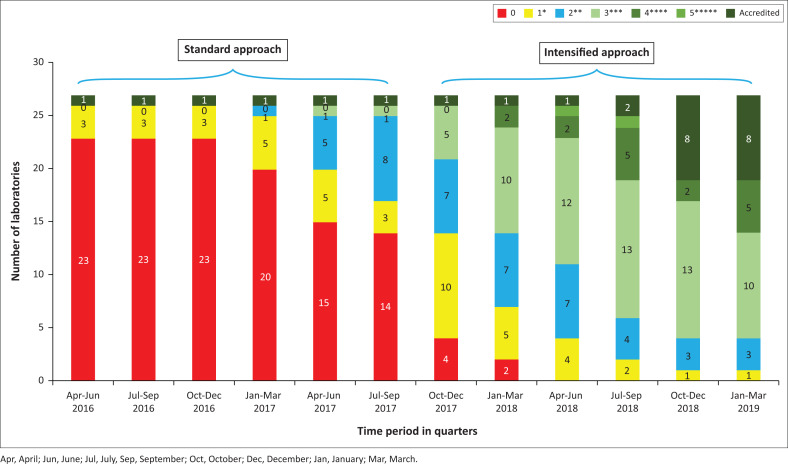
Audit star ratings comparison between the standard and intensified mentorship approach for University of Maryland, Baltimore-supported laboratories from April 2016 to March 2019, Kenya.

Eighteen months after implementing the intensified mentorship strategy (October 2017 – March 2019), eight laboratories were accredited, with five scoring four stars, 10 scoring three stars, three scoring two stars and only one laboratory scoring one star (Figure 4). Seven laboratories were newly accredited following the intensified mentorship, while the number of laboratories with ≥ 3 stars improved by 91%.

Across the evaluation period, the median SLIPTA checklist scores increased. With the standard mentorship approach, the median increased from 79 (interquartile range: 55–117) in April–June 2016 to 148 (interquartile range: 116–191) in July–September 2017. With the intensified mentorship, the median score increased to 225 (interquartile range: 207–275) by January–March 2019. The average change of audit scores while using the standard approach was 53.9. The average change with the intensified approach was 81.5, showing greater score improvement after implementing the intensified approach (Table 1).

**TABLE 1:** Median scores against the standard mentorship (April 2016 – Sep 2017) and the intensified mentorship approaches (Oct 2017 – Mar 2019) for University of Maryland, Baltimore-supported laboratories in Kenya.

Score period	Mean	s.d.	Median	p25	p75	Min	Max
April 2016 – June 2016	94.4	57.5	79	55	117	19	275
July 2017 – September 2017	148.3	48.8	148	116	191	70	275
January 2019 – March 2019	229.8	34.7	225	207	275	162	275

Note: Median = p50.

s.d., standard deviation; p25, 25th percentile; p75, 75th percentile; Min, minimum; Max, maximum.

## Discussion

The Boresha Maabara programme focused on intensified mentorship of facilities towards reaching and maintaining accreditation. This mentorship approach allowed facilities to be mentored by mentors with the skills necessary to close identified gaps. Mentors were allowed to focus on their areas of expertise, which led to a rapid improvement in laboratory audit scores and fast-tracked the laboratories towards accreditation.

The standard mentorship approach, as recommended by the SLMTA programme, assumes that knowledgeable and competent mentors capable of implementing all 12 Quality System Essentials independently are available.^13^ Makokha et al. noted that the standard mentorship approach that has embedded mentors handling all Quality System Essentials did not yield results in Kenya as expected, as the majority of the laboratories stagnated with minimal improvements.^12^ These findings suggest the need to re-strategise effective strategies towards laboratory accreditation.^12^ Each country may need to modify the standard approach and develop a country-specific strategy. Quality management system mentors may have varying skill levels across the different SLIPTA sections; therefore, they are naturally more effective mentoring in those areas of expertise. Our model of pairing QMS mentors based on their skills brought together a well-rounded team to provide mentorship to facilities and to resolve non-conformities quickly.

Other challenges of the standard approach have been documented, including unstructured and often unscheduled mentorship visits that lacked practical work plans and had inconsistent mentorship strengths; these circumstances led to an inability to implement some quality essentials as required.^13^ The intensified strategy adopted by UMB, led by an experienced QMS coordinator, provided structured mentorship visits by qualified mentors with monthly reviews of progress. Constant interactions between the QMS coordinator and mentors allowed opportunities to learn mentors individual strengths and weaknesses, thereby enabling suitable mentor pairs for a well-rounded team. Assigning mentors to facilities based on their skillsets and gaps identified in these facilities resulted in tasks being completed more quickly. Eventually, this model not only resulted in strengthened QMS in the facilities, but also in improved mentors’ technical capacities as mentors learned from each other. Monthly meetings between mentors and the QMS coordinator through virtual systems provided a forum for sharing progress and discussing laboratory-related challenges and allowed for follow-up of action items.

University of Maryland, Baltimore’s strategy also emphasised dedicating mentors solely to QMS, which allowed them to focus their time and effort towards mentoring assigned facilities towards accreditation. While this approach may appear resource-intensive, it allowed for shorter periods of intense interaction with facilities than the standard mentorship approach, as it enabled non-conformities to be efficiently resolved; the model is actually more beneficial for resource-limited countries.^14^ In our model, accrediting all facilities was in the best interest of all mentors; these mentors functioned together as a network and depended on each other rather than competing against each other.

Some challenges experienced in the field included inadequate laboratory staffing in the supported facilities; additional mentor-time spent on training lab personnel on QMS, as it is not part of the medical laboratory science training curriculum; lack of sufficient budget allocations for QMS activities; and use of substandard equipment failing the verification process. These challenges could be overcome by top management embracing QMS, building it as part of the medical laboratory science curriculum in the training institutions, and allocating portions of the budget specifically to QMS. These factors could also aid in maintaining the achieved gains over time.

### Limitations

The paper is restricted to UMB experience, staff, and mentorship structure in 10 Kenyan counties (Nairobi, Kiambu, Meru, Embu, Muranga, Nyeri, Laikipia, Nyandarua, Tharaka, and Kirinyanga) and is not necessarily representative of the whole country. The audits were also conducted by different people and are subject to inter-personnel variations.

### Conclusion

The intensified mentorship strategy, led by an experienced QMS coordinator working with QMS-dedicated mentor teams, resulted in accelerated progress towards accreditation. This strategy could be adopted to accelerate the process of accreditation in similar countries with proper planning and supervision to ensure success. Including top-level management, clinicians, and all laboratory staff in the QMS process is also essential for enhancing sustainability. Additionally, incorporating QMS as part of the medical laboratory science curriculum in the training institutions and allocating portions of the budget specifically for QMS could help maintain the achieved gains over time.
